# Cost-utility analysis in chronic kidney disease patients undergoing kidney transplant; what pays? A systematic review

**DOI:** 10.1186/s12962-020-00213-z

**Published:** 2020-05-19

**Authors:** Sameera Senanayake, Nicholas Graves, Helen Healy, Keshwar Baboolal, Sanjeewa Kularatna

**Affiliations:** 1 Australian Centre for Health Services Innovation, School of Public Health, Institute of Health and Biomedical Innovation, Queensland University of Technology, 60 Musk Ave, Kelvin Grove, Brisbane, QLD 4059 Australia; 2 Royal Brisbane Hospital for Women, Brisbane, Australia; 3 School of Medicine, University of Queensland, Brisbane, Australia

**Keywords:** Chronic kidney disease, Cost-utility analysis, QALY, Transplant

## Abstract

**Background:**

Health systems are under pressure to deliver more effective care without expansion of resources. This is particularly pertinent to diseases like chronic kidney disease (CKD) that are exacting substantial financial burden to many health systems. The aim of this study is to systematically review the Cost Utility Analysis (CUA) evidence generated across interventions for CKD patients undergoing kidney transplant (KT).

**Methods:**

A systemic review of CUA on the interventions for CKD patients undergoing KT was carried out using a search of the MEDLINE, CINAHL, EMBASE, PsycINFO and NHS-EED. The CHEERS checklist was used as a set of good practice criteria in determining the reporting quality of the economic evaluation. Quality of the data used to inform model parameters was determined using the modified hierarchies of data sources.

**Results:**

A total of 330 articles identified, 16 met the inclusion criteria. Almost all (n = 15) the studies were from high income countries. Out of the 24 characteristics assessed in the CHEERS checklist, more than 80% of the selected studies reported 14 of the characteristics. Reporting of the CUA were characterized by lack of transparency of model assumptions, narrow economic perspective and incomplete assessment of the effect of uncertainty in the model parameters on the results. The data used for the economic model were satisfactory quality. The authors of 13 studies reported the intervention as cost saving and improving quality of life, whereas three studies were cost increasing and improving quality of life. In addition to the baseline analysis, sensitivity analysis was performed in all the evaluations except one. Transplanting certain high-risk donor kidneys (high risk of HIV and Hepatitis-C infected kidneys, HLA mismatched kidneys, high Kidney Donor Profile Index) and a payment to living donors, were found to be cost-effective.

**Conclusions:**

The quality of economic evaluations reviewed in this paper were assessed to be satisfactory. Implementation of these strategies will significantly impact current systems of KT and require a systematic implementation plan and coordinated efforts from relevant stakeholders.

## Introduction

Chronic Kidney Disease (CKD) is a non-communicable disease and its burden is increasing globally [1]. At present countries round the world spend a significant proportion of gross domestic product on healthcare [2]. The exciting advances and innovations in medicine delivering superior patient outcomes often come at a higher cost. Fund holders are responding to the increasing financial stress by using the robust structural tools in the discipline of economic evaluation to guide budget decisions. Economic evaluation presents evidence to inform healthcare reimbursement decisions, particularly about value for money. It is used by the regulatory agencies of countries like Australia, United States and Switzerland in their evaluations of the cost-effectiveness of new health interventions prior to funding. The aim of these new healthcare investments should be the promotion of efficiency in resource allocation, not its degradation [3]. Cost-utility analysis (CUA) is generally the preferred method of economic evaluation that has been used to inform resource allocation decisions [4]. Compared to other economic evaluation method, CUA has the advantage of being able to incorporate patient reported outcomes and being able to compare a large number of potential outcomes included in the evaluation. The primary outcome in CUA is the ratio of change to total costs by change to total health benefits, measured by quality-adjusted life-years (QALYs). The incremental cost-effectiveness ratio (ICER) can be used to compare the value of different interventions [5] and the decisions are made within a constraint of the maximum “willingness to pay threshold (WTP)” for health benefits [6]. Different countries have adopted different WTP thresholds depending on the resources available [7].

Nowadays, non-communicable diseases pose a significant cost burden to health systems throughout the world. According to World Economic Forum, non-communicable diseases are ranked number one of the top global threats to economic development [8]. The Global Burden of Disease study attributes 2.17% (2.1%–2.2%) of deaths every year and 1.47% of disability-adjusted life years (DALYs) to CKD [9]. The increasing burden of CKD drives a pattern of growth in healthcare cost that is conflated. In 2012, $2.5 billion (1.7% of the total health expenditure) of direct healthcare costs funded by the Australian government were attributed to CKD [10, 11]. Most of the CKD related government expenditure was incurred by CKD patients at End Stage Kidney Disease receiving kidney replacement therapy. End Stage Kidney Disease function is incompatible with life and kidney replacement therapy is mandatory for patient survival [12]. The available kidney replacement therapy modalities include dialysis, either haemodialysis or peritoneal dialysis, and transplantation. Projections indicate that, in Australia the direct healthcare cost due to kidney replacement therapy will increase to $1.86 billion in 2020 ($1.09 billion in 2009) [13]. This growth in healthcare spending is not sustainable.

Kidney Transplantation confers the greatest utility and is the most cost-effective kidney replacement therapy modality compared to other kidney replacement therapies [13]. The financial benefits to the recipient, society and government of a successful kidney transplant (KT) are enormous. Maximising kidney transplantation rates is therefore a priority in cost effectiveness systems and End Stage Kidney Disease clinical programs. Both policy makers and clinicians use cost-effective outcomes when designing and implementing these systems and programs.

In the recent past novel strategies related to kidney transplantation have been introduced to healthcare market. However, many believe that novel strategies used in the kidney transplantation, such as strategies related to the transplantation itself or to post-transplantation practices, may consume additional resources. The former includes practices such as transplanting infectious kidneys (eg: transplanting Hepatitis C infected kidneys), kidney allocation practices (eg: payment to living donors) or different technologies used in KT (eg: pre-operative imaging using Digital Subtraction Angiography). The post-transplantation practices include practices such as use of different immunosuppression regimes. Since some of the novel strategies believed to be consuming resources, it is appropriate to evaluate the available cost-effectiveness evidence of different practices related to KT, to identify the ‘dominant’ (i.e.: cost saving and improves health) practices. In 2016, Jones-Hughes et al. conducted a comprehensive systematic review of the cost-effective studies related to different immunosuppression therapies [14]. However, cost-effective evidence of other KT related practices have not been reviewed adequately. A systematically conducted review of all the kidney transplant related CUA evidence will help the policy makers identify the most cost-effective interventions that produce best value for money. In this context, the aim of this study is to review the structure, the outcomes and the quality of published CUA evidence on different interventions available at the time of the kidney transplant for the CKD patients, using published quality appraisal checklists. The output of this effort would be a critical appraisal of currently available CUA evidence which would inform the policymakers and facilitate the dissemination and implementation of effective management strategies related to kidney transplant.

## Methods

### Search strategy

A systematic review was undertaken to identify all published studies relevant to cost utility analysi in CKD patients undergoing kidney transplant. The protocol has been published in Center for Open Science (OSF) (DOI : 10.17605/osf.io/xhywn) [15]. The review was conducted from October 2018 to March 2019 and initially all publications in English language published up to March 2019 were included in the review. However, later the search was updated to all published articles until March 2020.

Searches accessed the Medline, the Cumulative Index to Nursing and Allied Health Literature (CINAHL), EMBASE, PsycINFO and National Health Service Economic Evaluation Database (NHS EED) databases by using relevant key words (Additional file 1: Text box 1). The search included only the published journal articles. Previously published systematic reviews on CUA were used to identify the search terms [5, 16].

**Fig. 1:**
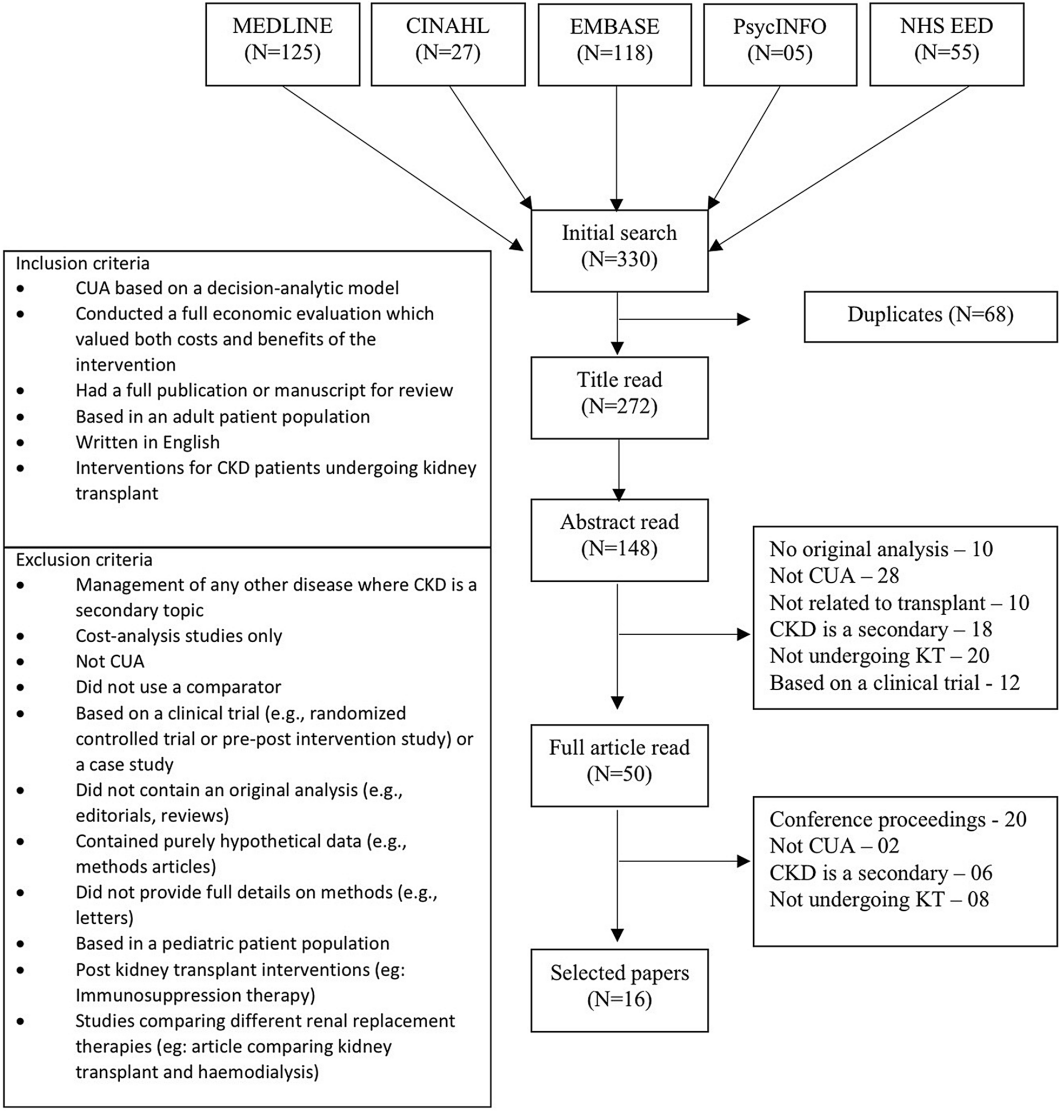
Inclusion and exclusion criteria and, PRISMA flowchart for the selection of articles for the review

Further, the reference lists of retrieved articles and review articles in this field of research were searched to identify additional published articles that met predefined inclusion and exclusion criteria (see Fig. 1). The review only focused on interventions in patients undergoing kidney transplant, thus studies of post kidney transplant patients and studies only compared different kidney replacement therapies were not included in the review.

### Data extraction

Data extraction was conducted by two independent reviewers (SS and SK) and discrepancies were resolved by discussion. Data fields extracted included research question, study population, setting and location, study perspective, intervention and the comparator, time horizon, discount rate, structure of the economic model, model assumptions, incremental cost and utility, sensitivity analysis—method and the results, characterizing heterogeneity, value of information analysis, Budget Impact Assessment and conclusions.

All monetary values were adjusted to 2016 USD by using CCEMG—EPPI-Centre Cost Converter (https://eppi.ioe.ac.uk/costconversion/). When this information was not reported, it was assumed to be 1 year before publication. The same US dollar value reported in the study was taken if the study was done after 2016. This adjustment is a two-stage process. In the first stage, using a Gross Domestic Product deflator index (‘GDPD values’), original estimate of cost from the original price year is converted to the target price year. In the second stage, the time adjusted cost estimate is converted from the original currency to a target currency, using conversion rates based on Purchasing Power Parities for GDP (‘PPP values’). Thus, this two stage process accounts for both inflation with in the country and fluctuation in exchange rates.

### Assessment of the quality of the economic evaluation

The CHEERS checklist was used as a set of good practice criteria for decision analytic modelling in determining the reporting quality of the economic evaluation. CHEERS check list has been increasingly used to assess the reporting quality of economic evaluations [17–19]. It has 24 criteria; assessing the title (01 criteria), abstract (01 criteria), background and objectives (01 criteria), methods (14 criteria), results (04 criteria), discussion (01 criteria) and other (02 criteria) (see Additional file 1: Table S1). Each item in the CHEERS checklist was scored as having met the criteria in full (“1”), not at all (“0”) or not applicable (NA). When items partially met the criteria, they were scored as “0”: no partial scores were assigned to avoid introducing subjectivity.

### Assessment of the quality of the data used in each evaluation

The quality of the data used to inform model parameters was determined using the modified hierarchies of data sources for economic analyses [20]. Each component of the decision model was assessed: baseline clinical data, costs and utilities. The quality of data sources was ranked from 1 to 4 (1 to 5 in cost data) with the highest quality of evidence ranked 1 (see Additional file 1: Table S2). For baseline clinical and cost data used in the evaluations, data from case series or analysis of reliable administrative data bases from the same jurisdiction were considered best quality evidence (rank 1), whereas expert opinion was considered the lowest quality evidence (rank 5 in baseline clinical data and rank 4 in costs data). For utility data used in the evaluations, either direct utility assessment for the specific study from a sample or indirect utility assessment for specific study from patient sample with the disease of interest were considered best quality evidence (rank 1) whereas utility valued derived from Delphi panels or expert opinion were considered the lowest quality (rank 4). In each of the component (i.e. baseline clinical data, costs and utilities), articles ranked either 1 or 2 were labeled as “High quality”, rank 3 as “Medium quality” and rank 4 or 5 as “Low quality”. This classification was carried out in accordance with the previously published literature [3]. For each article, the highest level of evidence used for each parameter was recorded.

Both assessment of the quality of the economic evaluations and assessment of the quality of the data used in each evaluation were conducted by two independent reviewers (SS and SK) and discrepancies were resolved by discussion.

## Results

A total of 330 articles were initially identified and reviewed and 16 met the inclusion criteria [21–36]. The reasons for the exclusion of 314 articles are described in Fig. 1 according to the PRISMA reporting guideline [37]. Analysis of the number of published economic evaluations that met the inclusion criteria for this review indicated an increasing trend in number of CUA published in medical literature. For descriptive statistical purposes the selected 16 articles were categorized in to three categories; CUA of transplanting infectious kidneys [26, 27, 34], CUA of kidney allocation policies [21–24, 29, 31, 33, 35, 36] and CUA of technology used in KT [25, 28, 30, 32].

### Assessment of the reporting quality of the economic evaluations

Table 1 shows how each of the 3 categories of articles in the review mapped to each criterion in the CHEERS checklist (see Additional file 1: Table S1). Out of the 24 characteristics, more than 80% of the selected studies reported 14 of the characteristics. The title clearly described the study as an economic evaluation only in 50% (n = 08) of the studies while a structured abstract with all the necessary information was reported in only 56.3% (n = 09) of the studies. The ‘choice of model’, which describes the specific type of decision analytic method used, ideally with a figure to show the model structure, and ‘model assumptions’ which describes all structural or other assumptions underpinning the decision-analytic model, were reported in 75.0% (n = 12) and 62.5% (n = 10) of the studies respectively.

**Table 1:** Quality scoring using the CHEERS criteria

	CHEERS criterion	CUA of transplanting infectious kidneys (n = 03)	CUA of kidney allocation policies (n = 09)	CUA of technology used in KT (n = 04)	Total (%) (n = 16)
1	Title	1	4	3	8 (50.0)
2	Abstract	1	5	3	9 (56.3)
3	Background and objectives	3	9	4	16 (100.0)
4	Target population and subgroups	3	9	4	16 (100.0)
5	Setting and location	2	9	3	14 (87.5)
6	Study perspective	2	8	4	14 (87.5)
7	Comparators	3	9	4	16 (100.0)
8	Time horizon	3	7	4	14 (87.5)
9	Discount rate	3	9	4	16 (100.0)
10	Choice of health outcomes	3	9	2	14 (87.5)
11	Measurement of effectiveness	2	9	4	15 (93.8)
12	Measurement and valuation of preference-based outcomes	NA	1^a^	NA	01 (100.0)
13	Estimating resources and costs	1	9	4	14 (87.5)
14	Currency, price date, and conversion	2	8	4	14 (87.5)
15	Choice of model	2	7	3	12 (75.0)
16	Assumptions	3	5	2	10 (62.5)
17	Analytical methods	0	5	0	05 (31.3)
18	Study parameters	2	6	4	12 (75.0)
19	Incremental costs and outcomes	3	8	3	14 (87.5)
20	Characterising uncertainty	3	8	4	15 (93.8)
21	Characterising heterogeneity	0	2	0	02 (12.5)
22	Study findings, limitations, generalisability, and current knowledge	2	7	4	13 (81.3)
23	Source of funding	1	7	2	10 (62.5)
24	Conflicts of interest	1	5	2	08 (50.0)

*NA* not applicable

^a^Applicable only to one study

### Assessment of the quality of the data used in evaluations

Table 2 describes the quality of the data used to inform model parameters was determined using the modified hierarchies of data sources for economic analyse (see Additional file 1: Table S2). The clinical, cost and utility data used in the three studies in the ‘transplanting infectious kidneys’ category were generally high quality (rank 1 or 2). The clinical and cost data had been gathered from reliable administrative databases or from published evidence from the same jurisdiction where the evaluation was conducted. Both clinical and utility parameters were of low quality (rank 4) in one of the studies in the above mentioned study category [27]. In both ‘CUA of kidney allocation policies, and ‘CUA of technology used in KT’, all the data sources used were generally considered to be of high quality (rank 1 or 2).

**Table 2:** Ranks of evidence for parameters used in the decision models

Evidence ranking	CUA of transplanting infectious kidneys (n = 03)			CUA of kidney allocation policies (n = 09)			CUA of technology used in KT (n = 04)		
	Clinical data	Cost data	Utility data	Clinical data	Cost data	Utility data	Clinical data	Cost data	Utility data
High quality									
Rank 1	1	1	–	8	8	1	–	2	–
Rank 2	1	2	2	1	1	8	4	2	4
Medium quality									
Rank 3	–	–	–	–	–	–	–	–	–
Low quality									
Rank 4	1	–	1	–	–	–	–	–	–
Rank 5	–	–	–	–	–	–	–	–	–

### Evidence of CUA

Almost all (15/16) [21, 22, 24–36] the studies included in the review were from high income countries [38], while only one was reported from lower-middle income countries (Malaysia) [23, 39]. Of the 15 reviews reported in the developed countries, seven studies were from United States (USA) and four were from Canada. Of the 16 studies included, three were related to CUA of transplanting infectious kidneys [26, 27, 34], nine were related to CUA of kidney allocation policies [21–24, 29, 31, 33, 35, 36] and four were related to CUA of technology used in KT [25, 28, 30, 32].

Table 3 provides an overview of the study characteristics of the reviewed models. Twelve studies were performed from a “healthcare payer perspective”, while five were from the societal perspective. The discount rate used ranged from 1.5 to 5% while the time horizon varied from 5 years to lifetime. Markov decision modelling was used in majority of the evaluations (13/16). Though the model structure used had not been explicitly mentioned, evidence of use of a Markov model was identified in two studies [23, 27].

**Table 3:** Summary of CUA of CKD patients undergoing kidney transplant included in the review

Study	Country	Year	Perspective	Discount rate (%)	Time horizon	Model structure
CUA of transplanting infectious kidneys (n = 03)						
Kadatz et al. [26]	Canada	2018	Health- care payer and Societal	1.5	10 years	Markov decision model
Kiberd et al. [27]	Canada	1994	Not explicitly stated (Health- care payer costs identified)	5	20 years	Not explicitly stated (Markov decision model identified)
Schweitzer et al. [34]	USA	2007	Societal perspective	3	20 years	Markov decision model
CUA of kidney allocation policies (n = 09)						
Axelrod et al. [21]	USA	2018	Health- care payer	3	10 years	Discreet Event Simulation
Smith et al. [35]	USA	2015	Health- care payer	3	20 years	Markov decision model
Mutinga et al. [31]	USA	2005	Not explicitly stated (Health- care payer costs identified)	5	20 years	Markov decision model
Schnitzler et al. [33]	USA	2003	Health- care payer	5	20 years	Markov decision model
Bavanandan et al. [23]	Malaysia	2015	Health- care payer	3	Life time	Not explicitly stated (Markov decision model identified)
Snyder et al. [36]	USA	2010	Societal perspective	3	10 years	Markov decision model
Cavallo et al. [24]	Italy	2014	Health- care payer	3.5	05 years	Markov decision model
Barnieh et al. [22]	Canada	2013	Health- care payer	5	Life time	Markov decision model
Matas et al. [29]	USA	2003	Societal perspective	5	20 years	Markov decision model
CUA of technology used in KT (n = 04)						
Nguyen et al. [32]	Australia	2015	Health- care payer	5	20 years	Markov decision model
McLaughlin et al. [30]	Canada	2006	Health- care payer	5	25 years	Markov decision model
Groen et al. [25]	Europe	2012	Health- care payer	4	10 years	Markov decision model
Liem et al. [28]	Netherlands	2003	Societal perspective	3	Life time	Markov decision model

*HCV NAT* Hepatitis C nucleic acid test, *CDC IRDs* Centers for Disease Control classified increased risk donors, *KDPI* Kidney Donor Profile Index, *ECD* expanded criteria donor, *SD* standard donor, LKT living kidney transplant, *DKT* deceased kidney transplant, *DBD* donation after brain death,*DCD* donation after cardiac death, *CDC* complement-dependent cytotoxicity

Table 4 provides the results of the CUA included in the review. The authors of 13 studies reported the intervention as cost saving and improving quality of life [21–28, 30, 32, 34–36], whereas three studies were cost increasing and improving quality of life [21, 24, 27]. Transplanting a Hepatitis C nucleic acid test positive deceased donor kidney followed by post-transplant direct acting anti-viral administration to the recipient, screening of all donors for HCV status and transplanting infected organs into HCV^+^ recipients, ignoring the HCV status of the donor when transplanting and transplanting kidneys from donors who are at increased risk of developing HIV or Hepatitis C were found to be cost-saving as well as delivering increased QALY. Further, several kidney allocation interventions were found to be cost-effective. Transplanting HLA 0‐3 or 4–6 mismatch live donor kidneys, a policy of transplanting the top 20% of the Kidney Donor Profile Index kidneys to candidates in the top 20% of expected survival, live donor transplantation, including both donation after brain death and donation after cardiac death kidneys in the allocation pool, the Programme Alba [40] implemented in Italy, a payment of US $8,000 (2010) to all the living donors which would expect the annual transplant rate to increase by 5%, were also found to be cost-effective. Using bead-based multiplex assays (threshold Mean Fluorescence Intensity level 500) with Complement-Dependent Cytotoxicity in screening for donor-specific anti-HLA antibodies to determine transplant suitability, use of flow screening only where patients’ immunological risks were stratified using the results of Flow Cytometry Cross Matching and flow micro-bead Panel Reactive Antibody when assessing the HLA antigen status, using hypothermic machine preservation as the organ preservation method in KT and pre-operative imaging of live kidney donors using Digital Subtraction Angiography were cost effective interventions. The intervention, HLA-B locus not matching before kidney allocation, was found to be cost saving while losing QALY [31]. The willingness to pay threshold (WTP) was reported in only two [21, 26] of the studies.

**Table 4:** Results of CUA of CKD patients undergoing kidney transplant included in the review

Study	Study population	Intervention	Comparator	Incremental cost effectiveness ratio (ICER)	Willingness to pay threshold	Sensitivity analysis-method	Sensitivity analysis-results
CUA of transplanting infectious kidneys (n = 03)							
Kadatz et al. [26]	Patients waitlisted for KT	Transplanting a HCV- NAT positive deceased donor kidney followed by post-transplant direct acting anti-viral administration	Remaining on the waitlist for a kidney transplant from an HCV NAT- negative donor	ICER is US$ 56,018 if receiving a HCV NAT positive kidney shortens the wait-time by 1 year. Remaining on the waitlist for 2 or more years is dominated^b^ compared to receiving a HCV NAT positive kidney	US $ 50,000	PSA, SA	Robust
Kiberd et al. [27]	Patients waitlisted for KT	Allocation polices based on donor and recipient HCV status	Comparison between each option	Option (b) over option (c)—ICER US$ 18,760/QALY. Option (a) over option (b)—Dominated^b^ Option (c) over option (a)—Dominant^a^	Not mentioned	SA	Variable
		discard all HCV^+^ donors					
		screen all donors and transplant infected organs into HCV^+^ recipients only					
		ignore HCV status and transplant without screening					
Schweitzer et al. [34]	Patients waitlisted for KT	Transplant kidneys from both standard donors and CDC-IRDs	Only transplant kidneys from standard donors. Discard kidneys from CDC-IRDs	Dominant^a^	Not mentioned	OW, SA	Robust
CUA of kidney allocation policies (n = 09)							
Axelrod et al. [21]	Patients waitlisted for KT	KDPI ≤85 DKT	Patients continuing on HD	US $ 83/QALY	US $ 100,000	Not done	–
		KDPI >85 DKT		US $ 32,870/QALY			
		PHS increased risk DKT		US $ 7944/QALY			
		HLA 0‐3 mismatch LKT		Dominant^a^			
		HLA 4‐6 mismatch LKT		Dominant^a^			
		ABOi LKT		US $ 34,755/QALY			
		ILKT		US $ 102,859/QALY			
Smith et al. [35]	Patients waitlisted for KT	A policy of transplanting the top 20% of the KDPI to candidates in the top 20% of expected survival	Conventional allocation policy	Dominant^a^	Not mentioned	OW	Robust
Mutinga et al. [31]	Patients waitlisted for KT	HLA-B locus not matched before kidney allocation	HLA-B locus matched before kidney allocation	US $ 7300 cost saving per lost QALY	Not mentioned	PSA, SA	Robust
Schnitzler et al. [33]	Patients waitlisted for KT	Accepting a ECD kidney	Accepting a standard kidney	ICER value not mentioned. SD US $ 56,058/QALY ECD US $ 72,838/QALY	Not mentioned	OW	Robust
Bavanandan et al. [23]	Patients waitlisted for KT	Kidney transplantation using live donors	Kidney transplantation using deceased donors	Dominant^a^	Not mentioned	OW	Robust
Snyder et al. [36]	Patients waitlisted for KT	A waitlist with both DBD and DCD kidneys	A waitlist only with DBD	Dominant^a^	Not mentioned	OW, TW, PSA	Robust
Cavallo et al. [24]	Patients waitlisted for KT	Assumption of 10 extra DCD transplants per year after implementing the programme Alba [40]	Baseline practice	US $ 7025/QALY	Not mentioned	OW	Variable
		Assumption of 10% extra transplants from each donation type (DCD, DBD, live) per year after implementing the programme Alba [40]	Baseline practice	Dominant^a^		OW	Variable
Barnieh et al. [22]	Patients waitlisted for KT	A payment of US $8000 (2010) to all the living donors, which would expect the annual transplant rate to increase by 5%.	Current KT practice	Dominant^a^	Not mentioned	OW, TW, PSA	Variable
Matas et al. [29]	Patients waitlisted for KT	Patients receiving a paid living unrelated donor kidney	Patients continuing on HD	It would be cost-effective to add one vendor to the donor pool if the payment made to that vendor for donation was no more than US $351,065	Not mentioned	OW	Variable
CUA of technology used in KT (n = 04)							
Nguyen et al. [32]	KT recipients (DKT and LKT)	Using bead-based multiplex assays (threshold MFI level 500) with CDC	Only CDC	Dominant^a^	Not mentioned	OW, PSA	Robust
McLaughlin et al. [30]	Patients undergoing DKT	Flow screening only, where patients’ immunological risks were stratified using the results of FCXM and flow micro-bead PRA	Serological screening only, where patients’ immunological risks were stratified using the result of AHG enhanced CDCXM and PRA titer only	Dominant^a^	Not mentioned	OW	Robust
Groen et al. [25]	Patients undergoing KT	Hypothermic machine preservation as the organ preservation method in KT	Use of Static cold storage	Dominant^a^	Not mentioned	Bootstrapping	Robust
Liem et al. [28]	Live kidney donors undergoing pre-operative imaging	Different combinations of strategies; MRIA, SCTA, DSA with MRA, MRIA and DSA if MRIA inconclusive, MRIA with SCTA	Pre-operative imaging DSA	DSA dominated all the imaging strategies	Not mentioned	OW, TW	Variable

*HCV NAT* Hepatitis C nucleic acid test, *HCV* Hepatitis C virus, *CDC IRDs* Centers for Disease Control classified increased risk donors, *KDPI* Kidney Donor Profile Index, *DKT* deceased kidney transplant, *LKT* living kidney transplant, *HD* Haemodialysis, *PHS* US Public Health Service, *ILKT* HLA incompatible living kidney transplant, *ECD* expanded criteria donor, *DBD* donation after brain death, *DCD* donation after cardiac death, *CDC* complement-dependent cytotoxicity, *MFI* mean fluorescence intensity, *FCXM* flow cytometry cross matching, *PRA* panel reactive antibody, *CDCXM* complement-dependent cytotoxicity crossmatch, *AGH* antihuman globulin, *MRI A* MRI Angiography, *SCTA* spiral CT angiography, *DSA* digital subtraction angiography, *PSA* probabilistic sensitivity analysis, *OW* on-way sensitivity analysis, *TW* two-way sensitivity analysis, *SA* scenario analysis

Dominant^a^—The intervention is cost saving and improves health compared to the comparator; Dominated^b^—The intervention is not cost saving and does not improves health compared to the comparator

In addition to the baseline analysis, sensitivity analysis was performed in all the evaluations except one [21]. Sensitivity analysis provides the information on the robustness of the baseline results according to different parameter estimates or, putting it another way, characterizes the effect of uncertainty in model parameters on the results [3, 41]. Of the evaluations which performed sensitivity analysis, 12 have performed deterministic sensitivity analysis [42], either one-way or two-way sensitivity analysis. Four studies [26, 27, 31, 34] have reported results of scenario analysis while one study [25] reported results of bootstrapping. Five evaluations that characterized parameters as distributions used probabilistic sensitivity analysis [22, 26, 31, 32, 36]. The results of the sensitivity analysis were robust in 15 of the evaluations, while the results were variable in five. In one-way, two-way or scenario analysis, if the baseline results do not significantly change or in probabilistic sensitivity analysis (PSA) if more than 50% of the iterations confirm the baseline results, the results of the sensitivity analysis was considered robust. Kadatz et al. [26] had gone one-step further and included information on Budget impact Assessment. However, none of the articles reported on Value of Information analysis.

## Discussion

This review systematically collated the published CUA studies on kidney transplantation. We reviewed existing model-based Cost utility Studies of the intervention kidney transplantation in CKD patients. Results indicate that transplanting certain high-risk donor kidneys (high risk of HIV and Hepatitis C virus (HCV) infected donor kidneys, HLA mismatched kidneys, kidneys with high Kidney Donor Profile Index) and a payment to living donors to be dominant strategies (i.e: cost saving and improves health). The reporting quality of the economic evaluations reviewed in this paper were found to be satisfactory.

### Quality of CUA

To assess the reporting quality of the economic evaluations, the 24 criteria in the CHEERS checklist were used as the benchmark good practice criteria for decision analytic modelling. According to the CHEERS guideline the title should reflect that the study is an economic evaluation and the abstract should provide a structured summary of the evaluation. The current review identified that the title and the abstract of most of the reviews selected was poor in quality. Similar findings have been found by Rosen et al. where it was found that the abstracts published in economic evaluations frequently omit information critical to proper study interpretation [43]. It is imperative that the title and the abstract contain all the necessary information because this is the only information accessible in some settings, especially in lower and middle income countries. In these jurisdictions health policy makers, planners and clinicians are forced to make decisions based on the subset of information in the abstract [44].

Perspective of the study is the viewpoint from which the intervention’s benefits and costs are evaluated. It is said that the societal perspective for economic evaluation is the ideal approach in assessing the profitability of societal investments (eg: kidney transplant) [45]. Eleven of the 16 studies included in the review used only healthcare payer’s perspective, five studies used societal perspective while only one study assessed both healthcare payers’ and societal perspective. Most of the interventions evaluated in the review not only incur cost to the healthcare payer but, in the border context, to the society as well (eg: productivity loss, premature deaths). Thus, narrow economic perspectives used in most of the evaluations limits the usefulness of these evaluations by excluding relevant costs and health outcomes from the analysis. Arguably for a chronic disease like CKD and its treatment the time horizon used in the model should be long enough to capture the chronic sequelae of the disease. Except Cavallo et al. [24], all the other studies had used 10 years or more as the time horizon and three studies have applied a life-time horizon for the CUA. However, these three studies have not explicitly stated the number of years that they have considered as “life-time horizon”. Considering the fact that mortality rates among post kidney transplant patients, even after a successful kidney transplant, is higher than the general population, explicitly stating the time horizon could be more informative.

Assumptions used in an economic model have a significant impact on model prediction [46]. The model assumptions were clearly reported in only 10 of the studies included in the review, which could adversely affect the transparency of some of the models used. Further, the level of confidence in the results of the economic evaluation can be boosted by sensitivity analyses. Of the several methods of sensitivity analyses, PSA provides the strongest evidence of robustness of the results, as it explicitly indicates the probability that an intervention is cost-effective [46]. When evaluating the results of this review, it is important to bear in mind that PSA has been performed only in five of the papers.

Stakeholders’ willingness to pay (WTP) threshold was reported only in two of the evaluations. WTP is an important characteristic that helps payers deciding how to weight resource allocation, in particular when interventions are more effective but more costly [47]. Value of information analysis, which estimates the added value of future cost-effectiveness research [48], Budget Impact Assessment, which assesses the financial consequences of adopting an intervention within a specific context and economic evaluations in decision making [49] are sophisticated methods that are gaining wider uptake. Value of information analysis was not reported in any of the evaluations reviewed while Budget Impact Assessment was reported in only one [26]. This limits the comprehensive understanding of the economic assessment of the new interventions discussed in this review.

The quality of data used in the models is an important factor when making a decision about the cost-effectiveness of one intervention over another. The quality of data used in the models is high. Most of the clinical and cost data were extracted from reliable data bases, which make the data sources high in quality. Almost all the studies used published data for the utility scores.

### Transforming CUA evidence in to practice

Though this study identified a substantial number of interventions for the KT population that appeared to be cost effective, it requires a robust systematic implementation to translate some of the interventions into practice. Currently safe and highly effective direct-acting antiviral (DAA) therapy is available for hepatitis C virus and recent evidence has demonstrated excellent safety and efficacy of DAA therapy in renal transplant recipients. Thus it is anticipated that recipients of HCV infected kidneys will have improved long-term graft and patient survival in the future [50–52]. Having said that, between 2005 and 2015 around 4000 donor kidneys have been discarded in United States for being positive for HCV, and it is expected that the numbers will fall after liberal use of DAA in the future [53]. Further, currently the recipient’s consent is needed to transplant a hepatitis C-positive organ and recipients’ willingness to receive infected kidneys has not been systematically evaluated. It is encouraging to note that the recent amendments to the United States’ law now permits people living with HIV to donate organs to HIV-infected recipients under research protocols [54]. This legislative effort has the potential to expand the donor pool by about 100 kidneys per year in the United States in the future [55].

Two of the studies in the reviews indicated that a payment to the living donors is a cost-effective strategy to increase the donor pool. Payment for organs is perhaps one of the most controversial interventions suggested to increase the kidney donor pool. It has generated disagreement on legal grounds and concerns about public acceptance [56]. At the core of the disagreements are moral and ethical concerns of commodification of the body and the risk for coercion leading to wealth-based distortions in decision-making [57–59].

Several kidney allocation policies (eg: a policy of transplanting the best quality kidneys to candidates with the highest expected survival, patients receiving a paid living unrelated donor kidney) were found to be cost effective in the review. But these allocations disadvantage certain groups eg elderly transplant candidates, fueling a debate about the equity of the new policies [60, 61]. Healthcare policy makers have particularly difficult decisions in this area, reconciling gain in quality of life associated with a specific change to allocation policy against any loss of equity to certain patient groups [56].

### Limitations

Though broad search strategies were used to find the relevant articles, we may have not identified all the model based economic evaluations reported in the specific area. Further, we found 20 abstracts presented in conference proceedings, in which the full article was not available for the review and were therefore not included. Our assessment of the reporting quality of the economic model and the quality of the data used in the model were based on the way the evaluations were reported rather than conducted. Thus, the conclusions should be interpreted with caution. Finally, an intrinsic limitation connected to assessment of quality (reporting quality and data quality) is that the results of the quality assessment may vary depending on the assessor. To minimize this potential bias, all studies were evaluated by the same two researchers and discrepancies were resolved by discussion.

## Conclusion

The reporting quality of the economic evaluations reviewed in this paper were assessed to be satisfactory. This systematic review of the cost utility analyses of kidney transplantation in CKD patients found that transplanting certain high-risk donor kidneys (high risk of HIV and HCV infected donor kidneys, HLA mismatched kidneys, kidneys with high KDRI) and a payment to living donors have the potential to be cost-effective strategies. The implementation of these strategies could significantly impact current systems of kidney transplantation and require sophisticated health service delivery, including a systematic implementation plan and coordinated efforts from relevant stakeholders.

## Supplementary information

**Additional file 1.** Additional textbox and tables.

## Data Availability

All data related to the study is available in the manuscript

## Abbreviations

CINAHL: Nursing and Allied Health Literature
CKD: Chronic kidney disease
CUA: Cost utility analysis
DAA: Direct-acting antiviral
DALYS: Disability-adjusted life years
HCV: Hepatitis C virus
HCV: Hepatitis C virus
HCV NAT: Hepatitis C nucleic acid test
ICER: Incremental cost-effectiveness ratio
KT: Kidney transplant
NHS EED: National Health Service Economic Evaluation Database
PSA: Probabilistic sensitivity analysis
QALYS: Quality-adjusted life-years
USA: United States
WTP: Willingness to pay threshold
