# Supplementary material for: Cost-utility analysis in chronic kidney disease patients undergoing kidney transplant; what pays? A systematic review
Source: Cost Eff Resour Alloc. 2020 May 19;18:18. doi: 10.1186/s12962-020-00213-z (PMC7236510; doi:10.1186/s12962-020-00213-z)
Supplement: Supplementary file 1 — Additional file 1. Additional textbox and tables. [file 12962_2020_213_MOESM1_ESM.docx]

# Additional material

| ***Text box 1: Search strategy used in each data base***   - “Chronic renal” OR "Chronic kidney" OR "Renal Insufficiency” OR “Kidney Failure” OR “Kidney Insufficiency” OR “Renal Failure” OR kidney OR renal   *and*   - Renal OR Kidney (in the title)   *and*   - QALY OR “Quality adjusted” OR “cost-utility” OR “cost-utility analysis” OR “cost-utility evaluation”   *and*   - transplant* |
| --- |

### Additional table S1 : Criteria used to assess the quality of the economic evaluation

| **Section** | **Criteria** |
| --- | --- |
| Title | Identify the study as an economic evaluation, or use more specific terms such as ‘‘cost-effectiveness analysis’’ and describe the interventions compared. |
| Abstract | Provide a structured summary of objectives, perspective, setting, methods (including study design and inputs), results (including base-case and uncertainty analyses), and conclusions |
| Background and objectives | Present the study question and its relevance for health policy or practice decisions. |
| Target population and subgroups | Describe characteristics of the base-case population and subgroups analyzed including why they were chosen |
| Setting and location | State relevant aspects of the system(s) in which the decision(s) need(s) to be made |
| Study perspective | Describe the perspective of the study and relate this to the costs being evaluated |
| Comparators | Describe the interventions or strategies being compared and state why they were chosen |
| Time horizon | State the time horizon(s) over which costs and consequences are being evaluated and say why appropriate. |
| Discount rate | Report the choice of discount rate(s) used for costs and outcomes and say why appropriate |
| Choice of health outcomes | Describe what outcomes were used as the measure(s) of benefit in the evaluation and their relevance for the type of analysis performed |
| Measurement of effectiveness | Synthesis-based estimates: Describe fully the methods used for the identification of included studies and synthesis of clinical effectiveness data. |
| Measurement and valuation of preference-based outcomes | If applicable, describe the population and methods used to elicit preferences for outcomes |
| Estimating resources and costs | Model-based economic evaluation: Describe approaches and data sources used to estimate resource use associated with model health states. Describe primary or secondary research methods for valuing each resource item in terms of its unit cost. Describe any adjustments made to approximate to opportunity costs |
| Currency, price date, and conversion | Report the dates of the estimated resource quantities and unit costs. Describe methods for adjusting estimated unit costs to the year of reported costs if necessary. Describe methods for converting costs into a common currency base and the exchange rate. |
| Choice of model | Describe and give reasons for the specific type of decision-analytic model used. Providing a figure to show model structure is strongly recommended. |
| Assumptions | Describe all structural or other assumptions underpinning the decision-analytic model |
| Analytic methods | Describe all analytic methods supporting the evaluation. This could include methods for dealing with skewed, missing, or censored data; extrapolation methods; methods for pooling data; approaches to validate or make adjustments (e.g., half-cycle corrections) to a model; and methods for handling population heterogeneity and uncertainty |
| Study parameters | Report the values, ranges, references, and if used, probability distributions for all parameters. Report reasons or sources for distributions used to represent uncertainty where appropriate. Providing a table to show the input values is strongly recommended |
| Incremental costs and outcomes | For each intervention, report mean values for the main categories of estimated costs and outcomes of interest, as well as mean differences between the comparator groups. If applicable, report incremental cost-effectiveness ratios |
| Characterizing uncertainty | Model-based economic evaluation: Describe the effects on the results of uncertainty for all input parameters, and uncertainty related to the structure of the model and assumptions |
| Characterizing heterogeneity | If applicable, report differences in costs, outcomes, or cost-effectiveness that can be explained by variations between subgroups of patients with different baseline characteristics or other observed variability in effects that are not reducible by more information |
| Study findings, limitations, generalizability, and current knowledge | Summarize key study findings and describe how they support the conclusions reached. Discuss limitations and the generalizability of the findings and how the findings fit with current knowledge |
| Source of funding | Describe how the study was funded and the role of the funder in the identification, design, conduct, and reporting of the analysis. Describe other nonmonetary sources of support |
| Conflicts of interest | Describe any potential for conflict of interest among study contributors in accordance with journal policy. In the absence of a journal policy, we recommend authors comply with International Committee of Medical Journal Editors’ recommendations |

### Additional table S2: Potential hierarchies of data sources for economic analyses

| *A - Baseline clinical data* | |
| --- | --- |
| 1 | - Case series or analysis of reliable administrative databases specifically conducted for the study covering patients solely from the jurisdiction of interest - Recent case series or analysis of reliable administrative databases covering patients solely from the jurisdiction of interest |
| 2 | - Recent case series or analysis of reliable administrative databases covering patients from another jurisdiction |
| 3 | - Old case series or analysis of reliable administrative databases Estimates from RCTs |
| 4 | - Estimates from previously published economic analyses: unsourced |
| 5 | - Expert opinion |
| *B - Costs* | |
| 1 | - Cost calculations based on reliable databases or data sources conducted for specific study – same jurisdiction - Recently published cost calculations based on reliable databases or data sources – same jurisdiction |
| 2 | - Unsourced data from previous economic evaluations – same jurisdiction - Recently published cost calculations based on reliable databases or data sources – different jurisdiction |
| 3 | - Unsourced data from previous economic evaluations – different jurisdiction |
| 4 | - Expert opinion |
| *C - Utilities* | |
| 1 | - Direct utility assessment for the specific study from a sample either:   (a) of the general population  (b) with knowledge of the disease(s) of interest  (c) of patients with the disease(s) of interest   - Indirect utility assessment for specific study from patient sample with disease(s) of interest; using a tool validated for the patient population |
| 2 | - Direct utility assessment from a previous study from a sample either:   (a) of the general population  (b) with knowledge of the disease(s) of interest  (c) of patients with the disease(s) of interest   - Indirect utility assessment from a previous study from patient sample with disease(s) of interest; using a tool validated for the patient population |
| 3 | - Indirect utility assessment from a patient sample with disease(s) of interest; using a tool not validated for the patient population Patient preference values obtained from a visual analogue scale |
| 4 | - Delphi panels, expert opinion |
